# Transcription-coupled recruitment of SMC complexes in yeast

**DOI:** 10.1093/femsyr/foag056

**Published:** 2026-10-21

**Authors:** Ji-Hyun Kim, Yo-Sub Chae, Kyoung-Dong Kim

**Affiliations:** Department of Systems Biotechnology, Chung-Ang University, Anseong 17546, Republic of Korea; Department of Systems Biotechnology, Chung-Ang University, Anseong 17546, Republic of Korea; Department of Systems Biotechnology, Chung-Ang University, Anseong 17546, Republic of Korea

**Keywords:** SMC complex, RNA polymerase, transcription, topological stress, loop extrusion, genome architecture

## Abstract

Despite their distinct biological functions, structural maintenance of chromosomes (SMC) complexes, including cohesin, condensin, and SMC5/6, preferentially accumulate at transcriptionally active loci, with recent studies revealing that SMC5/6 also shares the loop extrusion mechanism previously considered a hallmark of cohesin and condensin. Using yeast as a model system, this review discusses how RNA polymerases I, II, and III, together with their associated transcriptional machinery, contribute to the recruitment of SMC complexes to specific genomic sites. We examined the reciprocal interplay between transcription and SMC complexes, highlighting how transcription activity and SMC-driven chromatin organization influence each other. We further explore how SMC complexes recognize and respond to transcription-induced topological changes, such as positive supercoiling and R-loops. We propose that SMC complexes and transcription machineries form a dynamic regulatory network that coordinates the three-dimensional genome architecture with gene expression. Defining how each SMC complex is distinctly coordinated with RNA polymerase machineries and their associated factors, and how these complexes sense and resolve transcription-associated DNA topologies, represents an important frontier for future research.

## Teaser

This article explains how transcription machinery helps place SMC complexes on yeast chromosomes, linking gene activity with genome organization.

## Introduction

Structural maintenance of chromosomes (SMC) complexes comprise three distinct complexes—cohesin, condensin, and SMC5/6–each with a heterodimeric SMC ATPase. While each of these complexes performs specialized functions, such as sister chromatid cohesion, mitotic chromosome compaction, and genomic stability during DNA replication and repair, they share the fundamental ability to organize chromosomes through a common molecular mechanism known as loop extrusion, an ATP-dependent process in which they translocate along DNA to progressively enlarge chromatin loops and contribute to genome architecture.

Although cohesin, condensin, and SMC5/6 perform distinct chromosomal functions, their genome-wide localization patterns reveal a shared preference for transcriptionally active regions in yeast cells. Cohesin occupies centromeres (Glynn et al. 2004, Bernard et al. 2008), convergent transcription sites (Lengronne et al. 2004, Gullerova and Proudfoot 2008), and the rDNA array (Harris et al. 2014), which constitutes one of the most transcriptionally active regions in yeast due to intensive RNA polymerase I–mediated rRNA synthesis. Condensin is enriched at centromeres (Nakazawa et al. 2008, Snider et al. 2014), convergent genes (Nakazawa et al. 2015), rDNA (Wang et al. 2006, Nakazawa et al. 2008), tDNA (D’Ambrosio et al. 2008, Kim et al. 2016), and transcription termination sites of actively transcribed genes (Nakazawa et al. 2015, Sutani et al. 2015, Kim et al. 2016). SMC5/6 also accumulates at rDNA (Pebernard et al. 2008), telomeres (Pebernard et al. 2008), centromeres (Betts Lindroos et al. 2006, Pebernard et al. 2008, Outwin et al. 2009, Jeppsson et al. 2014), and highly transcribed genes (Roy et al. 2024). Thus, even though each SMC complex performs specialized roles, all three appear to be enriched in highly active chromosomal regions, supporting the idea that transcriptional activity may contribute to a common determinant of SMC localization.

Transcription in eukaryotes is mediated by three RNA polymerases, RNAP I, II, and III, each of which is responsible for different gene groups and operates in concert with dedicated transcription factors and co-regulatory complexes. These systems, together with their associated factors, transcription factors, and chromatin regulators, have emerged as key regulators of SMC complex localization to specific genomic loci. Once recruited, the SMC complexes do not passively occupy the chromatin. Instead, they engage in dynamic and reciprocal interactions with the transcriptional machinery, particularly RNAP II, enabling loop extrusion and reshaping of three-dimensional genome organization (Jeppsson et al. 2022, Guérin et al. 2024, Jeppsson et al. 2024, Lebreton et al. 2024, Chapard et al. 2025). Moreover, transcription is intrinsically linked to the generation of torsional stress in the chromatin. As RNAP moves along the DNA, the accumulated torsional stress at the active transcription sites serves as a signal for SMC complex localization (Jeppsson et al. 2024, Roy et al. 2024).

Given this context, this review uses the yeast system as the primary model to examine how the three SMC complexes are recruited to specific genomic loci in association with the RNA Polymerase I, II, and III (RNAP I, II, and III) transcription machineries, the mutual feedback dynamics between the transcription machinery and SMC complexes, and how SMC complexes are mobilized in response to transcription-induced topological stress.

## SMC complex recruitment at RNA Pol I and III-transcribed Loci

Ribosomal RNA genes (rDNA) are transcribed by RNAPI and are arranged as tandem repeats in the yeast genome. This genomic architecture creates a unique chromosomal environment in which exceptionally high transcriptional activity coincides with long repetitive DNA structures. As a result, the rDNA locus is particularly vulnerable to transcription-associated topological stress, unequal recombination, repeat copy number instability, and defects in mitotic condensation and segregation (Kobayashi 2011). SMC complexes play critical roles in maintaining the stability of rDNA repeats and ensuring their faithful segregation during mitosis (Freeman et al. 2000, Huang et al. 2006). Consistent with this notion, both condensin and cohesin are highly enriched at the rDNA locus in yeast, making the rDNA array a representative chromosomal region bound by SMC complexes *in vivo* (Glynn et al. 2004, Wang et al. 2006).

In budding yeast, Fob1 plays a central role in organizing the rDNA intergenic spacer into a regulatory platform that coordinates condensin recruitment and cohesin-associated recombination. Fob1 binds the replication fork barrier (RFB) within the non-transcribed spacer 1/intergenic spacer 1 (NTS1/IGS1) region of the rDNA repeat, where it promotes the association of rDNA regulatory factors, including the RENT complex components Net1, Sir2, Tof2, and cohibin, composed of Lrs4 and Csm1 (Kobayashi et al. 2004, Huang et al. 2006, Dinda et al. 2023). These factors contribute to rDNA silencing and recombination. In particular, Sir2 suppresses recombination between different rDNA repeats, thereby limiting rDNA copy number instability (Kobayashi et al. 2004). In addition, Tof2 and cohibin function with cohesin in a clamp-like mechanism at the rDNA recombination enhancer to inhibit homologous recombination (Freeman et al. 2000). The Fob1–Tof2–cohibin axis promotes condensin association with rDNA chromatin, supporting rDNA condensation, repeat segregation, and genome stability (Huang et al. 2006, Dinda et al. 2023). Thus, the Fob1-dependent assembly of RENT, Tof2, and cohibin at the RFB/NTS1 region links rDNA silencing and recombination suppression with two related but mechanistically distinct outcomes: cohesin-associated inhibition of homologous recombination and condensin-mediated rDNA condensation and segregation.

In contrast, condensin localization at rDNA in fission yeast appears to depend more strongly on rDNA chromatin architecture than on the Fob1–cohibin-centered mechanism (Tada et al. 2011). In *S. pombe*, Acr1 and Nuc1 bind to the upstream region of rDNA and contribute to the formation of a specialized rDNA chromatin structure that functions as a docking site for condensin accumulation (Nakazawa et al. 2008). Ark1/Aurora kinase and Cut17/Bir1/survivin further promote condensin localization to the rDNA and kinetochore regions (Nakazawa et al. 2008). Thus, although the detailed molecular factors differ between species, condensin enrichment at Pol I-transcribed rDNA commonly depends on specialized chromatin architecture and associated regulatory factors (Nakazawa et al. 2008, Tada et al. 2011, Dinda et al. 2023).

Transfer RNA genes (tDNAs) are dispersed throughout the chromosomes but are spatially clustered around the nucleolus in the nucleus (Haeusler et al. 2008). These loci are transcribed by RNAPIII, whose transcription machinery includes TFIIIC and TFIIIB. TFIIIC recognizes the internal promoter elements of tDNA, the A and B boxes, and promotes the subsequent assembly of TFIIIB and RNAPIII (Huibregtse and Engelke 1989, Kassavetis et al. 1989, Bartholomew et al. 1991). Beyond their role in Pol III transcription, tDNAs can influence local chromatin function, as their clustering and associated transcription machinery are linked to the transcriptional repression of nearby RNAPII promoters, a phenomenon known as tRNA gene-mediated silencing (Kinsey and Sandmeyer 1991, Hull et al. 1994, Kendall et al. 2000).

In budding yeast, TFIIIC is a key determinant of condensin recruitment to tDNA (Dubey and Gartenberg 2007, D’Ambrosio et al. 2008). Genome-wide ChIP analyses have shown that condensin-binding sites strongly overlap with tRNA genes and TFIIIC-bound sites (D’Ambrosio et al. 2008). Importantly, the B-box element recognized by TFIIIC is sufficient to form a minimal condensin-binding site, indicating that TFIIIC-bound promoter elements directly define condensin recruitment sites (D’Ambrosio et al. 2008). Consistently, co-immunoprecipitation analyses have shown that condensin associates with tRNA gene transcription complexes, including TFIIIB and TFIIIC, supporting a physical link between condensin and Pol III-associated factors (Haeusler et al. 2008).

A related connection between the Pol III transcription machinery and condensin has also been observed in fission yeast. In *S. pombe*, TATA-binding protein (TBP), a component of TFIIIB, contributes to condensin recruitment at transcriptionally active loci, in part, through its physical interaction with the condensin kleisin subunit Cnd2 (Iwasaki et al. 2010, 2015). Disruption of the Cnd2-TBP interaction reduces condensin’s localization at Pol III-transcribed loci and causes defects in mitotic chromosome organization and chromosome segregation (Iwasaki et al. 2010, 2015). Together, these findings suggest that the Pol III transcription machinery contributes to higher-order chromosome architecture by directing condensin to specific genomic loci.

Compared to condensin, the mechanism of cohesin recruitment at tDNA loci remains less clearly defined. Nevertheless, several observations suggest that the tDNA-bound Pol III transcription machinery contributes to cohesin function. In budding yeast, TFIIIC has been implicated in recruiting the Scc2/4 cohesin loader to tDNA regions, and tDNA elements, together with TFIIIB-associated factors, are required for the efficient establishment of cohesion at the neighboring HMR silent chromatin domain (Dubey and Gartenberg 2007, D’Ambrosio et al. 2008, Schmidt et al. 2009). Consistently, the HMR-proximal tDNA functions not only as a chromatin boundary element but also as an essential *cis*-acting element for cohesion establishment within the adjacent silent domain (Dubey and Gartenberg 2007). These findings suggest that Pol III-associated factors at tDNA can facilitate local cohesin loading or activation, although the underlying mechanism appears less direct than TFIIIC-dependent recruitment of condensin.

In contrast, a tDNA-specific role for SMC5/6 has not been clearly defined. SMC5/6 is instead best characterized at rDNA, where it contributes to replication-associated stability of the repetitive array (Peng et al. 2018). In budding yeast, rDNA repeats contain Fob1-bound RFB sites, which help minimize conflicts between 35S rRNA transcription and replication fork progression, but simultaneously create recurrent sites of programmed fork pausing (Kobayashi et al. 2004, Johzuka and Horiuchi 2007, Peng et al. 2018). Acute depletion of SMC5/6 causes defective rDNA replication and promotes the accumulation of Mph1-dependent recombination intermediates, including X-shaped DNA structures, at the RFB regions (Peng et al. 2018). Thus, SMC5/6 safeguards rDNA repeat stability by controlling the consequences of Fob1-dependent fork pausing and limiting Mph1-mediated recombination during rDNA replication (Peng et al. 2018).

Together, the rDNA and tDNA loci illustrate how RNA polymerase-associated chromatin environments contribute to chromosome organization through distinct SMC-related mechanisms. At tDNA loci, Pol III transcription factors provide a platform for condensin recruitment and may support local cohesin function. In contrast, at rDNA, Pol I-transcribed repetitive chromatin engages condensin, cohesin-associated factors, and SMC5/6 to coordinate condensation, recombination control, replication stability, and faithful inheritance of rDNA. An overview of these factors is presented in Table 1.

**Table 1:** RNAP machinery-associated factors that recruit SMC complexes across yeast.

RNA Polymerase	SMC complex	Key factor (organism)	Biological significance	Ref
RNAP I (rDNA)	Cohesin	Fob1, Tof2, cohibin(Lrs4/Csm1) (Sc)	rDNA repeat stability, suppression of unequal recombination, rDNA silencing	Kobayashi et al. 2004, Huang et al. 2006
	Condensin	Fob1, Tof2, cohibin(Lrs4/Csm1) (Sc)	rDNA condensation, faithful rDNA segregation, rDNA repeat stability	Freeman et al. 2000, Dinda et al. 2023
	Condensin	Acr1, Nuc1, Ark1/Aurora, Cut17/Bir1/Survivin (Sp)	rDNA condensation, faithful rDNA segregation, rDNA repeat stability	Nakazawa et al. 2008
	SMC5/6	Fob1 (RFB), Mph1 (Sc)	rDNA replication stability, suppression of fork-pausing-associated recombination	Peng et al. 2018
RNAP II	Cohesin	Mediator, RSC (Sc)	Sister chromatid cohesion, Chromatin accessibility for cohesin loading	Mattingly et al. 2022, Muñoz et al. 2022
	Condensin	Mediator, TBP (Sp)	Mitotic chromatin domain formation, chromosome segregation	Iwasaki et al. 2015, 2026
	Condensin	Ace2, Ams2, Sep1 (Sp)	Long-range network among cell cycle regulated genes	Nakazawa et al. 2015, Kim et al. 2016
	SMC5/6	SAGA (Sp)	Chromatin accessibility for SMC5/6 loading	Mahrik et al. 2023
RNAP III (tDNA)	Cohesin	TFIIIC, Scc2/4 (Sc)	tDNA-associated cohesion establishment	Dubey and Gartenberg 2007, D’Ambrosio et al. 2008
	Condensin	TBP, TFIIIC/TFIIIB-associated pol III machinery (Sp)	Pol III gene clustering/localization and mitotic chromosome organization	Iwasaki et al. 2010, 2015

## SMC complex recruitment by RNA Pol II-associated transcriptional regulators

Transcriptionally active chromosomal regions have emerged as important recruitment sites for SMC complexes in yeast. This transcription-coupled localization is mediated by open chromatin dynamics and, at least in part, by physical and functional interactions between SMC-associated factors and the RNAP II transcriptional machinery. The related factors are summarized in Table 1.

Among these interactions, the Mediator complex provides a particularly important molecular link between transcriptional regulation and SMC-dependent chromosome organization. Studies in yeast have shown that the Mediator contributes to the chromosomal recruitment of both cohesin in budding yeast and condensin in fission yeast, suggesting that it can function as a shared transcription-coupled anchoring platform for distinct SMC complexes (Mattingly et al. 2022, Iwasaki et al. 2026).

For cohesin to bind to chromosomes, the assistance of a loader complex (*S. cerevisiae*: Scc2/Scc4, *S. pombe*: Mis4/Ssl3) is essential, and the Scc2 subunit physically interacts with the Mediator complex (Mattingly et al. 2022). In budding yeast, depletion of the Mediator subunit Med14 causes defects in sister chromatid cohesion, the canonical function of cohesin, and ChIP-seq analysis showed a decrease in Scc2 binding at a subset of RNAP II-transcribed gene groups, particularly snoRNA and ribosomal protein genes (Mattingly et al. 2022). These results support a model in which the Mediator promotes the recruitment of the cohesin loader to these gene groups and facilitates cohesin loading.

Similarly, while cohesin forms a relatively short-range loop structure during interphase, condensin mediates long-range chromosome condensation during mitosis and shares this fundamental recruitment strategy (Tanizawa et al. 2017). Physical interaction between the condensin subunit Cnd1 and Mediator subunit Pmc4 has been identified, which serves as a driving force for recruiting condensin to highly transcribed gene loci and genes specifically activated during cell division (Iwasaki et al. 2026). Both the *cnd1*-K658E mutation (which inhibits the Cnd1-Pmc4 interaction) and *cnd1* deficiency impair condensin-mediated genomic contacts of 75-800 kb and the condensin domain. Experiments with Cnd1 and Pmc4 double depletion revealed that while the Mediator is required for condensin-dependent domain formation, it simultaneously plays a repressive role by suppressing condensin-independent genomic contacts within the ∼800 kb range (Iwasaki et al. 2026).

However, the recruitment of condensin to highly expressed genes is not mediated solely by the Mediator. It also strongly depends on the general transcription factor TBP. Condensin exhibits co-occupancy with TBP at highly transcribed RNAP II genes. This co-localization is driven by a direct physical interaction between TBP and the condensin subunit Cnd2 (Iwasaki et al. 2015). Mediator and TBP specifically bind to gene promoters, whereas condensin-binding peaks are generally positioned downstream. This suggests the potential for ongoing RNAP II transcription to reposition condensin along the gene body toward the 3’ end. In summary, factors such as the Mediator complex and TBP function as universal helpers for different SMC complexes across distinct cell cycle stages.

SMC complexes require accessible chromatin environments for efficient association with chromatin, and this accessibility is determined by chromatin remodelers and modifiers (Muñoz et al. 2019, 2022, Mahrik et al. 2023). In budding yeast, the RSC chromatin remodeling complex plays a central role in this process by maintaining nucleosome-depleted regions (NDRs) at promoters through its physical interaction with the Mediator (André et al. 2023). The RSC complex also supports RNAPII transcription initiation and elongation via direct interactions between its Rsc4 subunit and the RNA polymerase subunit Rpb5 (Soutourina et al. 2006). In this transcription-coupled chromatin environment, RSC cooperates with the cohesin loader Scc2–Scc4 to promote cohesin loading on chromatin (Muñoz et al. 2022). Mechanistically, RSC relaxes the chromatin structure, generates NDRs that serve as favorable substrates for cohesin deposition, and directly interacts with the Scc2–Scc4 loader through binding between Scc4 and a defined region of the RSC ATPase module (Muñoz et al. 2022). These findings suggest that RSC functions not only as a chromatin remodeler but also as a chromatin-associated platform that facilitates the recruitment and deposition of the cohesin loader.

Although SMC5/6 has been primarily studied in the context of DNA repair and replication-associated genomic regions, it is also associated with chromatin under steady-state conditions. In line with the idea that chromatin accessibility contributes to SMC complex recruitment, recent findings suggest that SMC5/6 loading is influenced by histone modifications. The histone acetyltransferase (HAT) module of the SAGA transcriptional coactivator complex physically interacts with SMC5/6 subunits, and the loss of its catalytic acetyltransferase subunit, Gcn5, reduces SMC5/6 occupancy specifically across gene regions (Mahrik et al. 2023). Consistently, reduced SMC5/6 occupancy in Δ*gcn5* mutants correlated with lower H3K9ac near transcription start sites and with the transcriptional activity of the affected loci. The acetyltransferase-impaired mutant *gcn5*-E191Q similarly decreased SMC5/6 occupancy at selected gene regions, supporting the role of Gcn5 catalytic activity in SMC5/6 chromatin association. However, the precise mechanism linking H3K9 acetylation and SMC5/6 recruitment remains unclear.

In addition to the basal transcriptional machinery and chromatin remodelers, specific transcription factors contribute to the spatial organization of SMC complexes, particularly condensin. In *S. pombe*, condensin is enriched at the target genes of the cell cycle regulators Ace2 and Ams2 (Kim et al. 2016). High-resolution Chromatin Interaction Analysis by Paired-End Tag sequencing (ChIA-PET) revealed that condensin forms a long-range chromatin interaction network that links Ace2/Ams2 target genes to centromeres. Consistently, loss of Ace2 reduces condensin occupancy at individual genes and disrupts the condensin-mediated long-range network among cell cycle-regulated genes (Kim et al. 2016). Condensin accumulation at mitotically active genes is also reduced in cells lacking the transcription factor Sep1 (Nakazawa et al. 2015). However, these transcription factors appear to promote condensin association through distinct mechanisms. Ace2 and Ams2 may directly recruit condensin, as both proteins co-immunoprecipitate with condensin and are sufficient to recruit condensin when tethered to an ectopic locus (Kim et al. 2016, Kim and Kim 2025). In contrast, Sep1 appears to act indirectly by driving the high-level transcription of mitotically upregulated genes. In the absence of Sep1, reduced transcription at these loci is accompanied by decreased condensin occupancy, suggesting that condensin association reflects RNAP II enrichment rather than direct recognition of Sep1. This interpretation is further supported by the observation that condensin accumulates at heat shock-inducible genes, specifically when they are strongly induced (Nakazawa et al. 2015).

Collectively, these studies indicate that SMC complexes are not recruited to the chromatin solely through genetic structural features. Instead, their genomic localization is shaped by a combination of chromatin accessibility, histone modifications, transcription-associated factors and sequence-specific transcriptional regulators. Through interactions with RNAPII-associated machinery, including the Mediator, TBP, RSC, SAGA, and specific transcription factors, SMC complexes are positioned at selected genomic regions, where they contribute to both local chromatin organization and higher-order three-dimensional genome architecture.

## Interplay between the transcriptional machinery and SMC-driven loop extrusion

SMC complexes and the RNAPII transcriptional machinery are functionally interconnected through a bidirectional relationship in which perturbation of one system can reshape both the three-dimensional chromatin organization and gene expression programs. This relationship has been confirmed across various cell cycle stages and experimental techniques, and the corresponding conditions are summarized in Table 2. This coupling is particularly evident for cohesin. Condensin also contributes specific functions, while the mechanism by which SMC5/6 influences transcription has yet to be elucidated. Defects in the cohesin loader or cohesin subunit broadly affect transcription throughout the cell cycle, and cohesin has been implicated in transcriptional regulation even during the G1 phase, before sister chromatid cohesion is established (Skibbens et al. 2010). Cohesin dysfunction is associated with reduced expression of genes with narrow nucleosome-depleted regions (NDRs), including genes involved in DNA damage responses and ribosome biogenesis, whereas genes associated with open chromatin and strong cohesin occupancy tend to be upregulated (Lindgren et al. 2014, Maya-Miles et al. 2019). Although the exact mechanism remains unclear, downregulated genes are associated with chromatin remodeling-dependent complexes, such as SAGA and Mediator, whereas upregulated genes are linked to TFIID and NuA4-driven transcriptional programs. This suggests that cohesin may differentially regulate transcription depending on the promoter chromatin architecture and transcriptional machinery employed (Maya-Miles et al. 2019). In *S. pombe*, cohesin also contributes to subtelomeric transcriptional homeostasis and promotes transcriptional termination at convergent genes, such that cohesin loss leads to read-through and overlapping transcript formation at these loci (Gullerova and Proudfoot 2008, Dheur et al. 2011).

**Table 2:** Summary of studies linking cohesin and condensin to RNAPII transcription by cell-cycle stage and species.

SMC complex	Species	Cell-cycle state	Perturbation	Evidence	Ref
**A. SMC complex → Transcription**					
Cohesin	*S. cerevisiae*	G1	*mcd1-1 (scc1)* ts mutant	*in vivo*—microarray	Skibbens et al. 2010
Cohesin	*S. cerevisiae*	G2/M	*scc2-4* ts mutant	*in vivo*—microarray, RT-qPCR	Lindgren et al. 2014
Cohesin	*S. cerevisiae*	Mitosis	*scc1-73* ts mutant	*in vivo*—RNA-seq	Maya-Miles et al. 2019
Cohesin	*S. pombe*	G1, G2	*rad21-K1* ts mutant	*in vivo–*3'RACE, TRO (Transcription Run-On)	Gullerova and Proudfoot 2008
Cohesin	*S. pombe*	G1	*mis4-367* ts mutant	*in vivo*—microarray, RT-qPCR	Dheur et al. 2011
Condensin	*S. pombe*	S, G2, Mitosis, Asynchronous	*cut14-208* ts mutant	*in vivo*—RT-qPCR, RNA-seq, ChIP-qPCR	Hocquet et al. 2018
Condensin	*S. pombe*	Mitosis	Cut14-AID	*in vivo*—RT-qPCR, northern blot, 3' RACE	Nakazawa et al. 2019
Condensin	*S. pombe*	Mitosis	Cnd2 shut-off (thiamine-repression)	*in vivo*—RT-qPCR, Immunoblotting	Sutani et al. 2015
Condensin	*S. pombe*	Mitosis	Cut14-AID, Cnd3-AID	*in vivo*—Hi-C	Kakui et al. 2017
Condensin	*S. cerevisiae*	Quiescence	SMC4-off (doxycycline-inducible tet-off depletion)	*in vivo*—Micro-C, ChIP	Swygert et al. 2019
**B. Transcription → SMC complex**					
Cohesin	*S. cerevisiae*	Mitosis	STE2 promoter deletion, HSP30 heat-shock induction	*in vivo*—ChIP-chip	Lengronne et al. 2004
Cohesin	*S. cerevisiae*	S, G2/M	*rat1-1* (transcription termination defect), *sua7-1* (gene looping defect)	*in vivo*—ChIP-chip, ChIP-qPCR, RT-qPCR	Ocampo-Hafalla et al. 2016
Cohesin	*S. cerevisiae*	S, G2/M	RNAPII anchor-away depletion, Estradiol treatment (GAL induction)	*in vivo*—Hi-C, ChIP-seq	Chapard et al. 2025
Cohesin	*S. cerevisiae*	S, G2/M	Thiolutin treatment	*in vivo*—Hi-C, ChIP-seq, ChIP-qPCR	Jeppsson et al. 2022
Cohesin	*S. cerevisiae*	G1, G2/M, Cell-cycle independent	*rat1-1* (transcription termination defect), RNAPII anchor-away, Scc3^3E^, Smc1^4E^	*in vivo*—micro-C, ChIP-seq*/in vitro*—single-molecule loop extrusion assay	Guérin et al. 2024
Condensin	*S. pombe*	Mitosis	*tfs1*ΔN overexpression (RNAPII backtracking), *sen1*Δ (RNAPIII termination defect)	*in vivo*—ChIP-qPCR	Rivosecchi et al. 2021
Condensin	*S. cerevisiae*	Asynchronous, G1, M	GAL On/Off	*in vivo*—Hi-C	Piveteau et al. 2026
Condensin	*S. pombe*	Mitosis	Rpb1-AID, Dhp1-AID	*in vivo*—ChIP-seq, spt-PALM, Hi-C	Lebreton et al. 2024

In contrast, condensins have a limited impact on global transcription (Hocquet et al. 2018, Nakazawa et al. 2019). Depletion of the condensin subunit Cnd2 in *S. pombe* results in only a modest reduction in the expression of condensin-bound genes (Sutani et al. 2015). However, condensin can still shape the transcription-associated chromosomal architecture in a context-dependent manner. In mitotic cells, condensin accumulates at the transcriptional termination sites of specific mitotically induced genes, potentially facilitating the release of persisting transcripts from chromosomes (Nakazawa et al. 2019). This allows the condensin to restrain local chromatin contacts, thereby changing the mitotic chromatin interaction landscape (Kakui et al. 2017). Moreover, the functional relationship between condensin and transcription becomes more pronounced in quiescent *S. cerevisiae* cells. In this state, condensin-dependent chromatin compaction helps maintain transcriptional repression, whereas condensin impairment leads to loop decompaction and genome-wide transcriptional activation (Swygert et al. 2019).

Transcriptional machinery plays a pivotal role in shaping the positioning of SMC complexes and in the progression of loop extrusion. Deficiency or impairment of RNAPII can reconfigure three-dimensional genome organization mediated by cohesin and condensin. The interaction between cohesin and RNAPII has been studied extensively in budding yeast. Cohesin is initially loaded near cohesin-loader binding sites and subsequently translocated toward the convergent terminator through RNAPII-dependent transcription elongation, where it accumulates at this position, which often functions as a loop anchor (Lengronne et al. 2004, Ocampo-Hafalla et al. 2016). Perturbation of RNAPII activity, such as termination failure in the *rat1-1* mutant or transcriptional activation upon GAL induction, leads to the repositioning of cohesin peaks and domain boundaries (Ocampo-Hafalla et al. 2016, Chapard et al. 2025). Similarly, inhibition of RNAPII by thiolutin weakens cohesin-associated loop boundaries and increases long-range cis interactions (Jeppsson et al. 2022) (Fig. 1A). Overall, these results support a model in which RNAPII functions as a semi-permissive moving barrier for cohesin.

**Figure 1:**
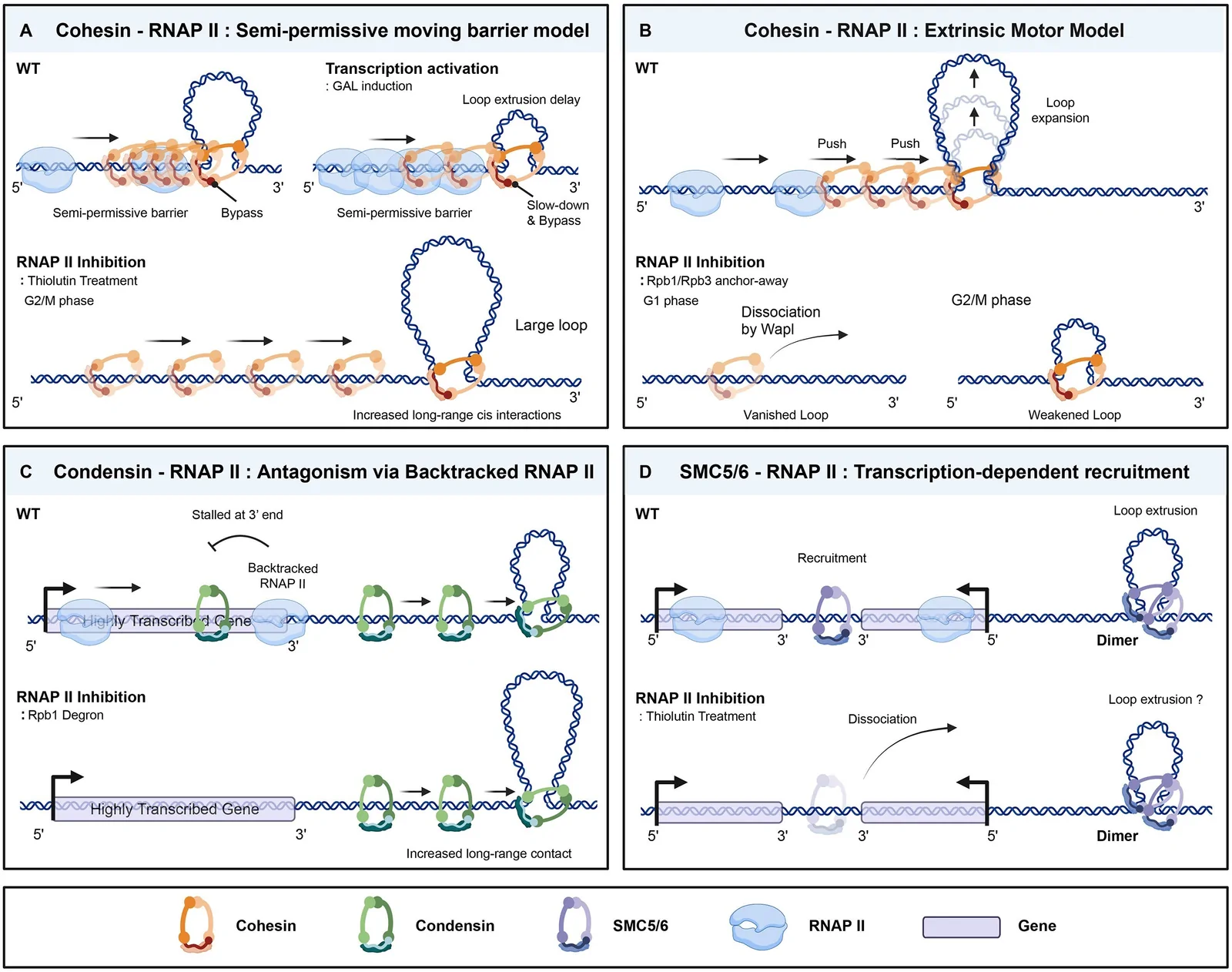
Distinct modes of SMC loop formation and localization upon RNAPII inhibition: a proposed model. (A) Cohesin–RNAPII: Semi-permissive moving-barrier model. RNAPII constrains cohesin extrusion as a movable roadblock: dense RNAPII limits loop extrusion, while reduced RNAPII permits larger loops. Wild type: RNAPII acts as a semi-permissive barrier. Transcription activation (GAL induction): High RNAPII occupancy generates denser permissive barriers, causing a delay in cohesin-mediated loop extrusion. RNAPII inhibition (Thiolutin treatment; RNAP initiation/elongation inhibition, causing global transcriptional inhibition): Loss of RNAPII barriers allows unconstrained extrusion, forming large loops and increasing long-range cis interactions. (B) Cohesin–RNAPII: Extrinsic-motor model. RNAPII acts as an extrinsic driving force that expands pre-existing loops. Wild type: Elongating RNAPII acts as an extrinsic motor that actively pushes cohesin along DNA, expanding pre-existing loops. RNAPII inhibition (Rpb1/Rpb3 anchor-away; rapamycin-induced nuclear export of intact RNAPII to cytoplasm): In G1, cohesin is removed by the cohesin release factor Wapl, leaving the loop undetectable (vanished loop). In G2/M, cohesin remains on chromatin but lacks the RNAPII motor, resulting in a weakened loop. (C) Condensin–RNAPII: Antagonism via Backtracked RNAPII. Wild type: Backtracked RNAPII present at the 3’ end of highly transcribed gene impedes condensin translocation, causing condensin to stall and accumulate at the 3’ end. RNAPII inhibition (Rpb1 degron; auxin-inducible direct proteasomal degradation of RNAPII subunit): Depletion of RNAPII, including the backtracked RNAPII, condensin translocates freely along chromatin, forming larger loops and leading to increased long-range contacts. (D) SMC5/6–RNAPII: Transcription-dependent SMC5/6 recruitment and dissociation model. Wild type: Active transcription generates positive supercoiling, especially at convergent intergenic regions, promoting SMC5/6 recruitment. The SMC5/6 dimer has a loop extrusion function. RNAPII inhibition (Thiolutin treatment; RNAP initiation/elongation inhibition, causing global transcriptional inhibition): Loss of persistent transcription-dependent topological changes, leading to the dissociation of SMC5/6 from chromatin. The effect of RNAPII inhibition on loop extrusion remains unknown. (This figure was created with BioRender.com.)

Although RNAPII is not strictly required for cohesin-dependent DNA loop formation, it promotes cohesin relocation toward convergent terminators while constraining the extent of loop extrusion through transcription-associated boundaries and active gene-associated long-range cis-contact (Chapard et al. 2025). Furthermore, studies using cohesin mutants with impaired loop-extruding capacity, such as Scc3^3E^ and Smc1^4E^, have proposed that RNAPII may act as an extrinsic motor that expands pre-existing loops (Guérin et al. 2024). Consistent with an active motor role, RNAPII anchor-away caused cohesin-mediated loop signals to vanish in G1, where cohesin dynamically turns over through the cohesin-releasing factor Wapl. In G2/M, where cohesin acetylation slows cohesin turnover, loop signals are weakened but remain detectable (Guérin et al. 2024). Recent high-resolution Hi-C and Micro-C analyses have further refined this model by examining whether RNAPII primarily serves as a barrier that constrains cohesin extrusion or as an active driver of loop formation (Guérin et al. 2024, Chapard et al. 2025) (Fig. 1B).

In contrast, studies on condensin-RNAPII interactions have revealed a more antagonistic relationship than that observed between cohesin and RNAPII. RNAPII backtracking dynamics and transcription termination are particularly important determinants of condensin chromatin occupancy and loop extrusion efficiency (Rivosecchi et al. 2021, Piveteau et al. 2026). In highly expressed genes, condensin typically accumulates near the 3’ end. This accumulation is driven by backtracked RNAPII, which physically impedes condensin bypass and traps the translocating condensin at the 3’ end (Fig. 1C). Similarly, transcription termination failure shifts the condensin peak toward the read-through direction (Rivosecchi et al. 2021). In budding yeast, loop extrusion by condensin transiently pauses immediately ahead of highly transcribed RNAPII genes, indicating that immediate consequences of RNAPII transcription on chromatin act as a roadblock for condensin (Piveteau et al. 2026).

Consistent with this antagonistic relationship, RNAPII dysfunction during mitosis reduces condensin occupancy at the 3’ end of gene. Under these conditions, condensin-mediated long-range contacts across domain boundaries are enhanced, with large mitotic domains being dissolved, suggesting that active RNAPII counteracts mitotic condensin-driven chromatin folding (Lebreton et al. 2024). More broadly, mitosis in fission yeast is accompanied by a global reduction in RNAPII activity, during which cohesin domains shrink, whereas large condensin domains expand (Tanizawa et al. 2017). These observations highlight the distinct RNAPII dependencies of cohesin and condensin.

In contrast, the loop extrusion capability of SMC5/6 has only recently been established, leaving its precise role unclear. Single-molecule imaging revealed that purified budding yeast SMC5/6 is an ATP-dependent loop-extruding motor in which the SMC5/6 dimer symmetrically enlarges DNA loops (Pradhan et al. 2023). However, the debate regarding the symmetry, asymmetry, and conformational states (such as monomer versus dimer) in the loop extrusion mechanism of SMC5/6 is still actively ongoing (Barth et al. 2025). Recent studies have suggested that this activity is related to the DNA topologies generated by transcription (Jeppsson et al. 2024). Consistently, thiolutin treatment causes the dissociation of SMC5/6 from chromatin, especially at centromeric and convergent intergenic regions, supporting a model in which ongoing transcription-dependent positive supercoiling promotes SMC5/6 recruitment as a topology-sensitive regulator (Fig. 1D).

Together, these findings indicate that the relationship between RNAPII and SMC complexes is not a simple one-dimensional interaction but rather a dynamic regulatory network that simultaneously shapes the three-dimensional genome architecture and transcriptional output.

## SMC complex responses to transcription-induced topological stress

As RNAPII moves along the DNA during transcription, it generates distinct forms of topological stress on either side of the transcription machinery. Positive supercoiling accumulates ahead of the advancing polymerase as the DNA double helix overwinds, whereas negative supercoiling develops behind it as the DNA unwinds and promotes R-loop formation (Fig. 2A). To resolve this stress, both type I and type II topoisomerases act on different aspects of this stress by relaxing transcription-induced supercoiling (Jeppsson et al. 2024). Top1 is also involved in restraining R-loop accumulation, and loss of Top1 accordingly promotes R-loop accumulation at highly transcribed, G-rich sequences (Yadav et al. 2016). Top2, unlike Top1, additionally performs sister-chromatid decatenation, a distinct activity that also links it to condensin function (Charbin et al. 2014). Rather than being merely passive byproducts of gene expression, these transcription-induced topological structures are increasingly recognized as potential cues for the localization and function of SMC complexes in the cell. In this context, individual SMC complexes may exploit the distinct biochemical features of these abnormal DNA structures to recognize target sites and execute specialized chromatin functions.

**Figure 2:**
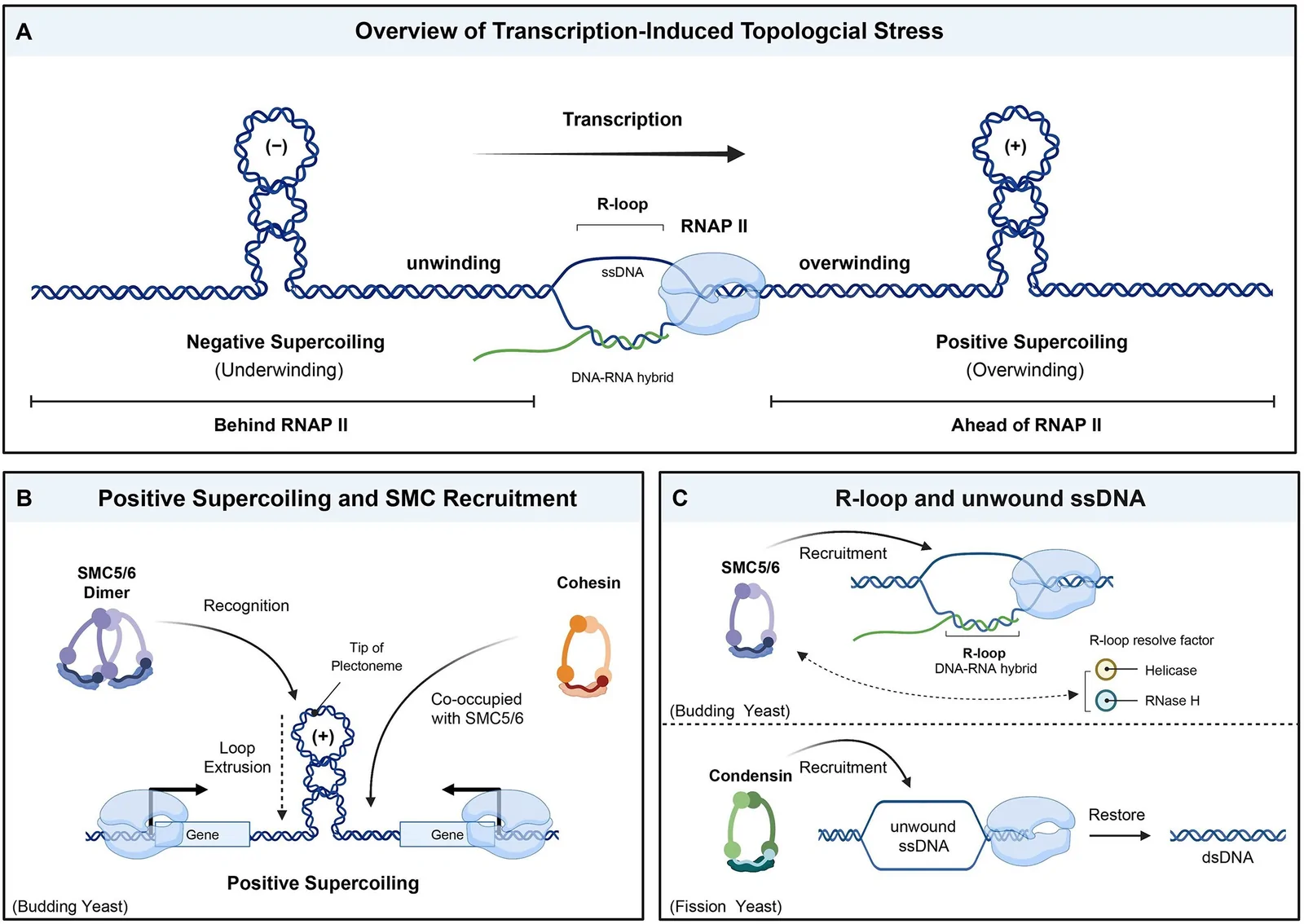
Transcription-coupled topological stress shapes SMC complex recruitment in yeast: a proposed model. (A) Overview of Transcription-induced topological stress. Transcription by RNAPII generates positive supercoiling (overwinding) ahead of the polymerase and negative supercoiling (underwinding) behind it. The unwound DNA behind RNAP II promotes R-loop formation, a three-stranded structure in which the nascent RNA hybridized with the template DNA strand, displacing the non-template strand. (B) Positive Supercoiling and SMC recruitment. In budding yeast, SMC5/6 dimer recognizes the tip of positively supercoiled DNA plectonemes, generated by convergent RNAPII transcription, and initiates loop extrusion. And cohesin co-occupies these loci. (C) R-loop and unwound ssDNA. (Top) In budding yeast, SMC5/6 is recruited to R-loops. These R-loops are resolved by factors, including helicase and RNase H. SMC5/6 genetically interacts with these factors. (Bottom) In fission yeast, Condensin is recruited to unwound ssDNA regions, generated at actively transcribed genes during mitosis. Condensin promotes DNA renaturation, restoring unwound ssDNA to dsDNA. (This figure was created with BioRender.com.)

The SMC5/6 complex appears to be the most direct sensor of transcription-associated topological stress. Its preferential association with positively supercoiled DNA strongly influences chromosomal distribution. In budding yeast, SMC5/6 dimers preferentially recognize the tips of positively supercoiled DNA plectonemes and initiate loop extrusion from these structures (Jeppsson et al. 2024). This topological preference may explain the prominent accumulation of SMC5/6 at convergent genes, where opposing RNAPII transcription units are expected to generate elevated positive supercoiling (Fig. 2B). *In vivo*, SMC5/6 accumulates at convergent genes only when Top1 and Top2 are inhibited simultaneously, implicating transcription-induced positive supercoiling (Jeppsson et al. 2024). Cohesin also overlaps with SMC5/6 at supercoiled regions and convergent genes (Betts Lindroos et al. 2006, Jeppsson et al. 2014, 2024). Consistently, genome-wide GapR-seq, which maps positively supercoiled DNA through the selective binding of the bacterial protein GapR, revealed a spatial overlap between positive supercoiling and cohesin binding sites in *S. cerevisiae* (Guo et al. 2021). These observations suggest that positive supercoiling marks chromosomal regions where both cohesin and SMC5/6 accumulate. However, the relationship between the two complexes is not reciprocal: cohesin dysfunction reduces SMC5/6 occupancy during the G2/M phase, whereas SMC5/6 dysfunction does not substantially affect cohesin binding (Outwin et al. 2009, Jeppsson et al. 2014). Thus, although cohesin and SMC5/6 occupy related topologically stressed loci, SMC5/6 localization appears to depend, in part, on cohesin rather than the reverse.

In addition to its association with positively supercoiled DNA, SMC5/6 has been linked to R-loop structures in highly transcribed genes. R-loops are three-stranded nucleic acid structures formed when nascent RNA hybridizes with the DNA template strand, displacing the non-template DNA strand. These structures can accumulate when RNAPII pauses or stalls, particularly under conditions of replication stress, DNA damage, or transcription-replication conflicts, and are normally resolved by helicases, topoisomerases, RNase H, and mRNP biogenesis factors (Aguilera and García-Muse 2012, Allison and Wang 2019). Genome-wide studies have shown that R-loops are frequently enriched at highly transcribed genes, telomeres, and DNA replication fork-associated regions (Chan et al. 2014, Lafuente-Barquero et al. 2017), many of which coincide with SMC5/6-binding sites (Roy et al. 2024) (Fig. 2C). Consistent with this spatial association, biochemical analyses using purified human SMC5/6 core complex showed that SMC5/6 can bind short RNA-DNA hybrid structures *in vitro*, suggesting that the complex has an intrinsic capacity to recognize nucleic acid structures associated with R-loop-containing chromatin (Serrano et al. 2020).

Genetic screening in *S. cerevisiae* further supports the functional interactions between SMC5/6 and RNA-DNA hybrid metabolism. SMC5/6 subunits genetically interact with factors involved in R-loop resolution or processing, including RNase H enzymes such as Rnh1 and Rnh203, the RNA-DNA helicase Sen1, and Rat1, which contributes to the processing or removal of telomeric repeat-containing RNA (Roy et al. 2024). This connection is particularly relevant to replication forks challenged by R-loop-associated stress. SMC5 physically interacts with the Mph1 helicase (Chen et al. 2009), and SMC5/6 has been proposed to restrain excessive Mph1 activity, thereby limiting the accumulation of toxic recombination intermediates at sites where the R-loop impedes replication fork progression (Lafuente-Barquero et al. 2017). Related functions appear to be at least partially conserved in human cells, where SMC5/6 preferentially associates with positively supercoiled DNA *in vivo* and contributes to the resolution of transcription-replication conflicts (TRCs), which can otherwise promote R-loop accumulation and genome instability (Wu et al. 2026). Together, these findings suggest that SMC5/6 responds to transcription-associated topological and R-loop-mediated stress by recognizing RNA-DNA hybrid-associated chromatin and stabilizing replication forks in vulnerable genomic regions.

Condensin also responds to transcription-associated DNA topology, although its recruitment mechanism appears distinct from that of SMC5/6. Rather than primarily recognizing pre-existing supercoiled structures, condensin in fission yeast preferentially associates with single-stranded, unwound DNA regions generated at actively transcribed genes during mitosis. Depletion of condensin leads to the accumulation of unwound ssDNA at these sites, suggesting that condensin may help resolve transcription-generated DNA unwinding (Sutani et al. 2015). Consistent with this idea, the condensin core subcomplex Cut3-Cut14 has been proposed to promote DNA renaturation, thereby eliminating the unwound DNA intermediates produced by transcription. This activity may facilitate the subsequent establishment of global positive supercoiling across mitotic chromosomes, contributing to chromosome condensation (Sutani et al. 2015). Thus, whereas SMC5/6 appears to recognize pre-formed positive supercoiled DNA, condensin in yeast may act at an earlier topological intermediate by binding unwound DNA and remodeling it toward a more condensed, positively supercoiled state (Fig. 2C).

A conceptually related mechanism has also been proposed for cohesin, although the direct evidence is derived from a replication-based rather than a transcription-based context. Bubble DNA, mimicking an unwound state, promotes topological loading of cohesin *in vitro* (Murayama et al. 2024). Because transcription also transiently exposes unwound DNA and ssDNA, a recent unified model proposes that transcription-exposed ssDNA may serve as a substrate for cohesin capture, although this possibility remains to be tested (Uhlmann 2025). Recent *in vivo* evidence supports the notion that cohesin performs loop capture at transcription-induced unwound DNA (Guérin et al. 2024). These observations suggest that unwound DNA may represent a broader topological cue for SMC complex loading or activation, however, the evidence currently differs among SMC complexes and biological contexts.

Biochemical studies further support the idea that condensin can actively remodel DNA topology. Although condensin-mediated supercoil generation has not been directly confirmed in yeast, vertebrate condensin I can induce positive supercoils into relaxed circular DNA in an ATP-dependent manner *in vitro* (Kimura and Hirano 1997, Kimura et al. 2001). Single-molecule imaging has further shown that condensin preferentially loads onto positively supercoiled plectonemes and actively generates supercoils in an ATP-dependent manner (Kim et al. 2022). However, these results were derived using an artificially supercoiled DNA substrate that cannot distinguish between the topological stress resulting from transcription and replication. Therefore, it remains unclear whether transcription-induced positive supercoiling specifically promotes condensin recruitment and activation *in vivo*. Nevertheless, these findings indicate that condensin is not merely a passive responder to DNA topology but has the capacity to actively remodel DNA structures in a manner that may contribute to chromosome condensation and resolution. Condensin also cooperates functionally with Top2 *in vivo*. Condensin is required for the completion of Top2-mediated sister-chromatid decatenation (Charbin et al. 2014), and its loop extrusion is proposed to minimize Top2-mediated DNA knots, demonstrating a capability distinct from cohesin and SMC5/6 (Dyson et al. 2021). This functional cooperation is further supported by genetic interactions, as a condensin mutant displays synthetic lethality with a *top2* mutant (Robellet et al. 2014). These interactions are well characterized in the context of mitotic segregation and DNA replication, and whether they extend specifically to transcription-induced topological stress remains unclear.

Together, these observations underscore a broader principle of SMC biology: SMC complexes can recognize specific DNA topologies generated by transcription, yet their functions may extend beyond passive sensing to active remodeling of these structures. How SMC complexes distinguish among positive supercoils, unwound DNA, R-loops, and other transcription-associated intermediates, and whether their recruitment is coupled to accessory factors that promote structural resolution, remain important questions for future investigation.

## Conclusion

In this review, we systematically report the mechanisms by which the three SMC complexes are recruited to specific locations by transcriptional machinery, such as RNAP I, II, and III or associated factors, the reciprocal dynamics between SMC complexes and RNAP II, and the response of SMC complexes to transcription-induced torsional stress in yeast. A series of studies illustrated that transcription collaboratively contributes to the loop extrusion efficiency and three-dimensional chromosome structure of the SMC complex through active recursive feedback, rather than a passive factor. Cohesin recruitment via the Mediator and RSC, cohesin repositioning during RNAPII elongation, and loop anchoring at convergent genes constitute a closely interconnected sequential process. Condensin is driven by transcription factors such as Ace2 and Ams2 to mitotic active genes, and backtracked RNAPII acts as a barrier to condensin loop extrusion. Finally, SMC5/6 binds to chromatin under normal conditions via the SAGA complex and specifically recognizes transcription-induced supercoiled structures to initiate loop extrusion.

However, significant challenges remain unresolved. The precise role of SMC5/6 in collaboration with the transcriptional machinery has been studied much less than that of cohesin and condensin, and the molecular details of how SMC5/6 directly cooperates with or competes against the transcriptional machinery to influence loop extrusion remain largely unclear. Similarly, while the three SMC complexes bind to similar locations, our understanding of the crosstalk between these complexes, specifically how cohesin, condensin, and SMC5/6 coordinate or compete with one another at the same genomic location, remains rudimentary. Despite evidence that in eukaryotic systems, condensin functions by excluding or bypassing cohesin during chromosome formation in mitosis, the dynamic relationship among the three complexes during interphase or other specific conditions remains poorly understood.

Integrated approaches, such as single-molecule imaging, high-resolution chromatin contact mapping, and conditional protein inactivation approaches (rapid degron systems, anchor-away, and chemical inhibition) (Haruki et al. 2008, Nishimura et al. 2009, Qiu et al. 2024), are expected to enable research that elucidates the previously unknown interactions mentioned above. Such studies will ultimately shed light on evolutionarily conserved principles of genome organization that apply from yeast to mammals, with fundamental implications for understanding gene regulation, chromosome segregation, and genome stability in both health and disease.
